# Modulating Immune Response with Nucleic Acid Nanoparticles

**DOI:** 10.3390/molecules24203740

**Published:** 2019-10-17

**Authors:** Jake K. Durbin, Daniel K. Miller, Julia Niekamp, Emil F. Khisamutdinov

**Affiliations:** Department of Chemistry, Ball State University, Muncie, IN 47306, USA; jdurbin@bsu.edu (J.K.D.); dkmiller2@bsu.edu (D.K.M.); jmniekamp@bsu.edu (J.N.)

**Keywords:** nucleic acid, nanotechnology, immune response

## Abstract

Nano-objects made of nucleic acids are becoming promising materials in the biomedical field. This is, in part, due to DNA and RNA self-assembly properties that can be accurately computed to fabricate various complex nanoarchitectures of 2D and 3D shapes. The nanoparticles can be assembled from DNA, RNA, and chemically modified oligonucleotide mixtures which, in turn, influence their chemical and biophysical properties. Solid-phase synthesis allows large-scale production of individual oligonucleotide strands with batch-to-batch consistency and exceptional purity. All of these advantageous characteristics of nucleic-acid-based nanoparticles were known to be exceptionally useful as a nanoplatform for drug delivery purposes. Recently, several important discoveries have been achieved, demonstrating that nucleic acid nanoparticles (NANPs) can also be used to modulate the immune response of host cells. The purpose of this review is to briefly overview studies demonstrating architectural design principles of NANPs, as well as the ability of NANPs to control immune responses.

## 1. Introduction

The human immune response can be classified as innate, adaptive, or both. The innate response is nonspecific, and the response rate is usually rapid, thus making it the body’s ‘first line of defense’. On the other hand, the adaptive response is an acquired response that is more specific and involves memory. With adaptive immunity, each successive exposure to the foreign substance increases the defensive response of the immune system, but innate immunity is present prior to any exposure to the foreign body. Both pathogen-associated molecular patterns (PAMPs) and damage-associated molecular patterns (DAMPs) are known to induce immune responses, and they have been extensively studied for medicinal applications [1,2]. In PAMPs, evolutionarily conserved and diverse chemical identities of peptides, nucleic acids, oligonucleotides, lipids, lipoproteins, and polysaccharides are expressed by a wide variety of infectious microorganisms that trigger the activation of innate immunity. The recognition of PAMPs are mediated by pattern recognition receptors including toll-like receptors (TLRs), which are the largest and most extensively studied classes of pattern recognition receptors [3,4]. The innate immune response promoted by TLR activation is characterized by the production of proinflammatory cytokines [5]. Cytokines are a small family of proteins that coordinate the immune and inflammatory responses of innate and adaptive immune systems. Interleukins, tumor necrosis factors, hematopoietic growth factors, and interferons are some examples of cytokine types. Subsequently, these proinflammatory factors signal the host to start production of high affinity antibodies in order to generate cytotoxic T cells to fight infections [6].

The ability to control the immune system response to foreign substances is extremely challenging due to the broad and diverse chemical identities of molecular patterns. The nano-objects made of nucleic acids are highly attractive candidates in the developmental immunology field. Immunomodulation therapy using nucleic acid material is an emerging field [7,8,9,10,11] and offers a new approach to treat diseases by enhancing, inducing, or suppressing an immune response to benefit the host. Programmable self-assembly properties of RNA and DNA allows for the design of various intricate architectures exemplified by recently developed nucleic acid polygons [9,12,13], nano-cubes [14,15], prisms [16], nano-rings [17,18], and other objects including three-way [19,20,21] and four-way junctions [22]. Another advantage of such nucleic acid nano-particles (NANPs) is that their individual oligonucleotide strands can be chemically synthesized enabling large-scale production, batch-to-batch consistency, and exceptional purity. Furthermore, they can be assembled from DNA, RNA and chemically modified RNA mixtures [23,24], which, in turn, influence their biophysical and chemical properties, including stability in blood serum [25], thermodynamic stability [26], geometrical appearances [27] and most importantly, immunological properties [9]. The ability of NANPs to induce the host immune response is only on the verge of discovery and promises to play a critical role in nanomedicine.

This review focusses on discussion of geometrical properties of naturally occurring RNA structural motifs and artificial DNA and RNA building blocks that are commonly used to fabricate various intricate nano-objects of 2D and 3D shapes of no more than 50 nm. Furthermore, their application as immunomodulators is explained with emphasis on immunostimulatory cytosine-phosphate-guanosine (CpG) oligonucleotides, as well as other approaches, including NANPs with specific shapes, sequences, and nucleic acid composition affecting immune cells *in vitro*.

## 2. Design of RNA Nanoparticles from Naturally Occurring Structural Motifs.

### 2.1. RNA Three Way and Other Multiway Junctions.

Hypothetically, any naturally occurring RNA motifs can be implemented to construct an RNA complex of a specific shape. This is becoming more evident as a broad class of different RNA motifs was implemented in nanoparticle design and now play a critical role in the assembly of RNA nano-architectures. A few of those examples include kissing hairpin loops [28], cognate hairpin loop/loop–receptor pairs [29], paranemic motifs [30], the right-angle motif [31], kink-turns [32,33], C-loops [34], multi-helix junctions [35] and protein binding motifs [36,37]. Perhaps, the A-form RNA double-helix is the central building block as it provides a regulatory element to support and combine other RNA modules in 3D space.

**Figure 1:**
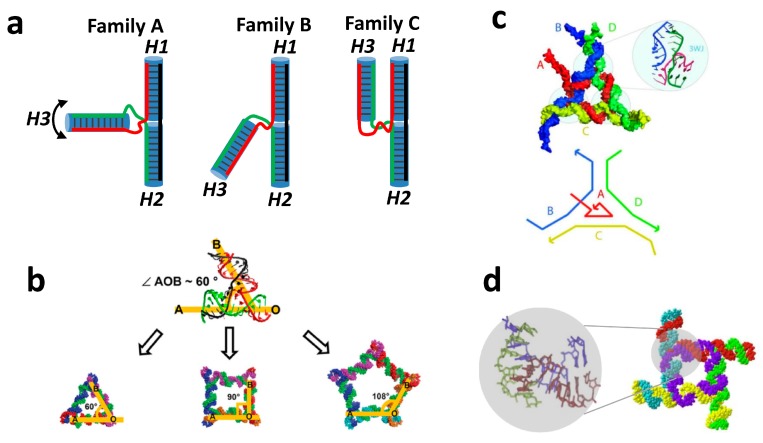
Examples of NANPs fabricated from RNA 3WJ. (**a**) Three RNA 3WJ families A, B, and C are differentiated based on the location of the Helix 3. (**b**) RNA polygons assembled from pRNA 3WJ, adapted with permission from Ref [13]. Copyright 2014 Oxford University Press. (**c**) RNA triangle assembled from 3WJ obtained from RNAJunction database accession ID 11836, adapted with permission from Ref [41]. Copyright 2011 American Chemical Society. (**d**) Planar RNA square nanoconstruct assembled from 3WJ found in large ribosomal subunit PDB I 2OGM, adapted with permission from Ref [42]. Copyright 2011 American Chemical Society.

Another well-known RNA structural motif routinely used to fabricate various shapes is the RNA three-way junction (3WJ), which consists of three interconnected RNA strands [21]. According to Westhof’s work on investigation of crystal structures of RNA 3WJ motifs in various large RNA crystallographic structures, the overall geometry can be divided into three major families of the 3WJ including A, B, and C, as exemplified in Figure 1 [35]. In these examples, coaxial stacking is always observed between Helix 1 and Helix 2, and Helix 3 can employ flexible angles depending on the family type. One of the most commonly used RNA 3WJ motifs, belonging presumably to the A type 3WJ, is from the central domain of Phi29 pRNA. The geometrical properties of this motif have been extensively studied to fabricate various shapes of 2D and 3D nanoparticles as a carrier of regulatory RNAs by Peixuan Guo’s laboratory [13,16,21,38,39]. The 3WJ motif is flexible and can stretch its angle from 60° to 108° to form planar polygonal nanoparticles, as exemplified by Khisamutdinov et al. [13] (Figure 1b). The individual RNA strands typically contain 2′-fluoro modifications on ribose moiety of pyrimidines (2′f-U/C) to elevate stability in blood serum and retain pharmacokinetics and pharmacodynamics properties. The assembled planar triangular structures based on the 3WJ motif can be further used to assemble structures of 3D triangular nanoprisms to load and carry medicinal compounds [16].

Various geometrical 3WJ motifs can be extracted from the RNA junction database developed in Shapiro’s group, and this software allows particular 3WJ family characteristics to be searched and organized [40]. One of the examples was demonstrated by construction of an equilateral triangle. The specific search for a 3WJ exhibiting features of B family with an angle of 60° between H2 and H3 was utilized to computationally design and experimentally validate the assembly of equilateral triangles [41] (Figure 1c). Similarly, the same approach was used to locate a 3WJ with a 90° angle to construct square-shaped nanoparticles [42].

Application of another naturally occurring 3WJ motif from 23S rRNA of *H. marismortui* was demonstrated by Jaeger’s group, where the tetragonal shape RNA nanoparticle named tectosquares was assembled [43]. More impressively, various structural motifs, including 3WJ and 5WJ, were used by the same group to assemble intricate structures of other tect-otriangles, tecto-squares, and RNA nano-hearts [44].

### 2.2. RNA Kink-Turn Motif and Other Helical Bends.

**Figure 2:**
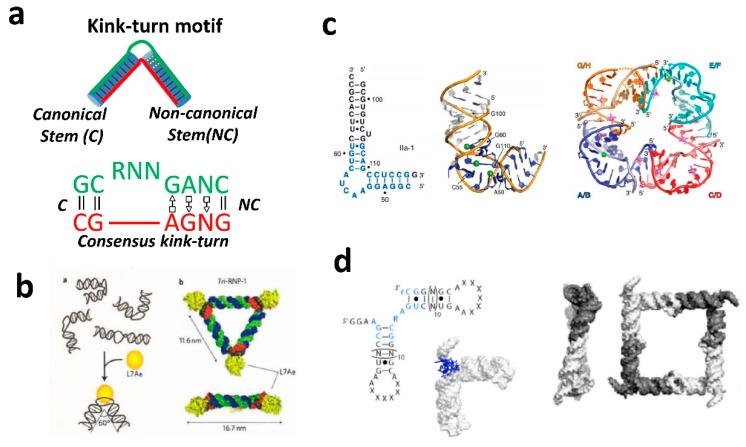
Nano-constructs assembled using motifs that create bends and an RNA helix. (**a**) The kink-turn motif is a well-studied RNA motif that forms flexible and rigid angular conformations in large RNA molecules. (**b**) RNA equilateral triangle assembled using the protein-binding properties of the kink-turn motif. Figure 2b was adapted with permission from [36], Copyright 2011 Springer Nature. Examples of RNA motifs forming 90° angles that were utilized to assemble tetragonal nanoparticles from the RNA motif of (**c**) subdomain IIa of IRES and (**d)** right-angle motif from ribosomal RNA. Figure 2c,d were adapted with permission from ref# [53] (Copyright 2011 PNAS) and ref# [43] (Copyright 2009 American Chemical Society) respectively.

The kink-turn (k-turn) motif is an architectural motif that was shown to be applicable to design and assemble various shapes, including triangular DNA and RNA structures. The kink turn motif is schematically demonstrated in Figure 2. The motif was first visualized by analysis of the *Haloarcula marismortui* large ribosomal subunit [33,45]. This motif comprises an internal loop in double-stranded RNA (dsRNA) that introduces a very tight kink into the helical axis [33]. The motif is a critical structural element in ribosomal RNA. K-turn motifs are present six times in *H.marismortui* 23S rRNA, and they are also prominent in the structure of *Thermus thermophiles* 16S rRNA [33]. These K-turn motifs also appear in the structures of U4 snRNA and L30e mRNA fragments [46]. The K-turn is a two-stranded, helix-internal loop-helix motif comprised of approximately 15 nucleotides. The “canonical stem” (C-stem) is the first helical stem which ends at the internal loop with two Watson–Crick base pairs, typically C–G base pairs. The “non-canonical” stem (NC-stem) is the second helical stem which follows the internal loop and starts with two non-Watson–Crick base pairs, typically G–A base pairs (Figure 2a) [47]. The internal loop between the helical stems is always asymmetrical and usually has three unpaired nucleotides on one strand and none on the other. Most of the K-turn examples in the ribosome are involved in protein binding, indicating this motif is an important protein recognition element able to engage in multiple intermolecular interactions simultaneously [48]. The structural features of kink turns have been extensively studied by the Lilley group [49]. One of the examples to implement the kink turn motif is to assemble a quazi-cyclic RNA complex by associating two, three, or four motifs [47]. To demonstrate that the kink-turn motif can be used in complexation with a protein to form RNA nanoparticles of different shapes, Saito’s group used protein-binding properties of the kink-turn motif [50,51,52]. Binding of ribosomal protein L7Ae induces a conformational change of the RNA motif to create a 60° angle, resulting in the formation of equilateral triangles [36] (Figure 2b). Other motifs with a predefined angular geometry include: ligand-responsive RNA switch module extracted from subdomain IIa of the IRES element of Seneca Valley virus RNA [53], right-angle motif obtained from ribosomal RNA [31], and complex structural motif from tRNA [43]. The structural features of the above RNA motifs were often used within 2D shapes, such as triangle and square nanoparticles (Figure 2).

### 2.3. RNA Kissing Loop Motifs and Loop–Receptor Interaction.

**Figure 3:**
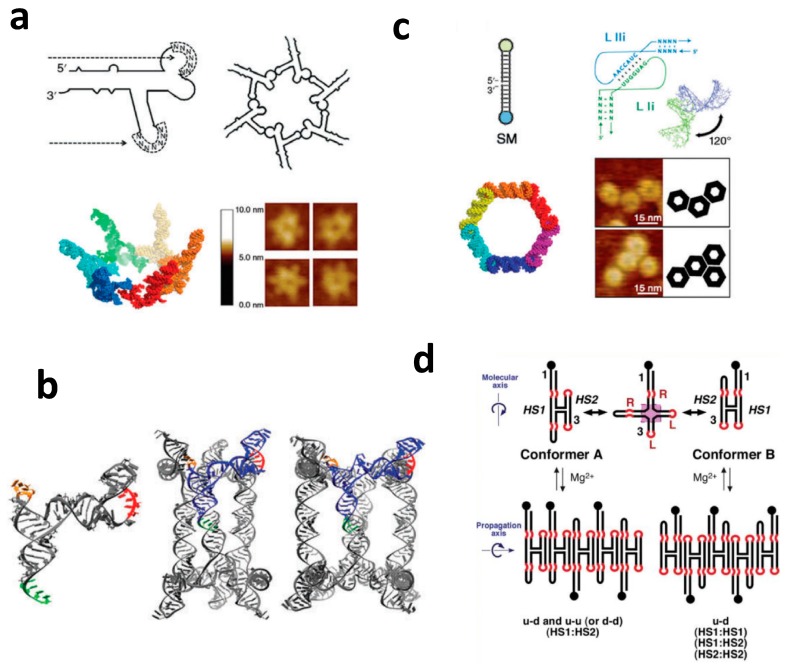
RNA nanostructures assembled from loop–loop and receptor–loop interactions. (**a**) Examples of pRNA assembly into hexamers and (**b**) RNA nanocages facilitated by pRNA loop–loop interactions. (**c**) Nanoring structures obtained by using advantage of 120° angle formed by kissing–loop interactions between RNAI and RNAII loop sequences of ColE1. (**d**) Examples of RNA filaments self-assembled via intermolecular receptor-loop loop-receptor interactons. Figure 3a–d were adapted with permissions from Ref [54] (Copyright 2013 RNA Society), Ref [55] (Copyright 2014 Springer Nature), Ref [18] (Copyright 2011 American Chemical Society), and [57] (Copyright 2006 Oxford University Press), respectively.

Assembly of multimeric RNA nano-objects of predefined shapes can also be achieved using specific hairpin loop–hairpin loop (kissing–loop complex) or internal loop/loop–receptor interaction motifs that can be combined in pairs for high affinity. Such interacting motifs are important components and often used to combine multiple building blocks into multimeric RNA nanoparticles of globular or planar shapes. For example, the previously mentioned pRNA from the phage phi29 DNA packaging motor have been shown to assemble into multimeric pRNA nanoparticles, as well as 3D RNA triangular and tetragonal nanoprisms by utilizing a bottom-up self-assembly principle [28,54,55] (Figure 3a,b). Such shape versatility offers various combinations of aptamers and ribozymes, which can be attached to enhance the functionalization of these structures to enable entrance into the cell. Assembly of multimeric RNA nano-complexes using kissing–loop complexes were also assembled from the inverse of the RNAI and RNAII loop sequences of ColE1 plasmid-encoded transcripts [18,56]. The bend of 120° in the RNA module between adjacent RNA helices provides an ideal angle to assemble hexameric nanostructures, termed nanorings (Figure 3c). Leontis and coworkers demonstrated rational design of RNA filaments utilizing internal loop/loop–receptor interaction motifs [29,57]. The pairs of internal GNRA loop/loop–receptor interaction motifs were used to engineer RNA monomers that self-assembled into straight, micrometer-long filaments.

## 3. Design of RNA Nanoparticles from Computationally Designed Structural Motifs.

With the previously mentioned design principles, several variations and modifications to the nanoparticle shapes can be employed using computationally developed RNA building blocks. In this approach, the design principles of RNA nanoparticles folding into pre-programmed configurations is dictated by the formation of A-form RNA helices *via* canonical Watson–Crick base-pairing. The design relies on the energy minima driven by the formation of the RNA/RNA duplex with the assumption that any potentially formed 3D configuration supported by non-canonical interactions will have unfavorable free-energy formation. Overall, RNA complexation is driven by helical folding and the helix can be treated as a rigid module. This design principle is known as a helix-centric approach [15] and has been utilized to form nanoparticles made of RNA, DNA and hybrid RNA/DNA nanoparticles [8,9,24]. In the helix centric approach, secondary structure folding algorithms encompassed within *mfold* can be effectively engaged [58]. This allows selection of particular RNA or DNA sequences that can form double-stranded helixes with varying thermodynamic parameters.

RNA 3D nanocubes were one of the earliest examples of nanoparticles designed using the helix-centric approach [14,15]. Due to their smaller size, the uracils were used as helical linkers to connect dsRNA helixes resulting in intricate RNA 3D nanoobjects. Furthermore, utilizing the potential flexibility of four sequential uridines and thymidines, a broad spectrum of 2D DNA and RNA structures were constructed [9].

All previously mentioned examples of nucleic acid nanoparticles have demonstrated tremendous potential in nanomedicine and have shown to be potent nanovehicles to carry RNA functional moieties such as aptamers, proteins, riboswitches, ribozymes, or short interfering RNAs (siRNAs) to target and treat cancer cells [13,59]. However, there are only limited reports available that demonstrate how these structures can affect immunological properties of a potential host cells. In general, there are two main strategies that are used to stimulate immune response. The first and most common way is to employ immunostimulatory oligonucleotides [60,61,62,63], and the second is to use various NANPs as immunostimulators, depending on their shapes, sequences, and nucleic acid composition [8,10,11]. Below, we briefly describe both approaches.

### 3.1. NANPs as Carriers of Immunostimulatory CpG Oligonucleotides.

In the field of therapeutic nucleic acids, unmethylated cytosine-phosphate-guanosine (CpG) oligodeoxynucleotides (ODNs) have received tremendous attention [13,64,65,66,67]. Unmethylated dinucleotides, cytidine and guanosine, appear frequently in prokaryotic DNA but are rare in eukaryotic DNA [68]. When eukaryotic cells are infected with bacterial DNA, they initiate expression of TLR-9 to these CpG sequences and activate a protective immune response [69,70,71,72]. The immunostimulatory activity of bacterial DNA can be mimicked by synthetic oligodeoxynucleotides that have these CpG repeats [73]. However, the immediate recognition of CpG by immune cells often triggers a vigorous stimulation of the immune system, resulting in uncontrollable and often severe inflammatory responses [74,75]. Due to the advantage of programmable NANPs, the selective attachment of various numbers of synthetic CpG ODNs to nanoparticles is possible, thus controlling undesirable responses.

**Figure 4:**
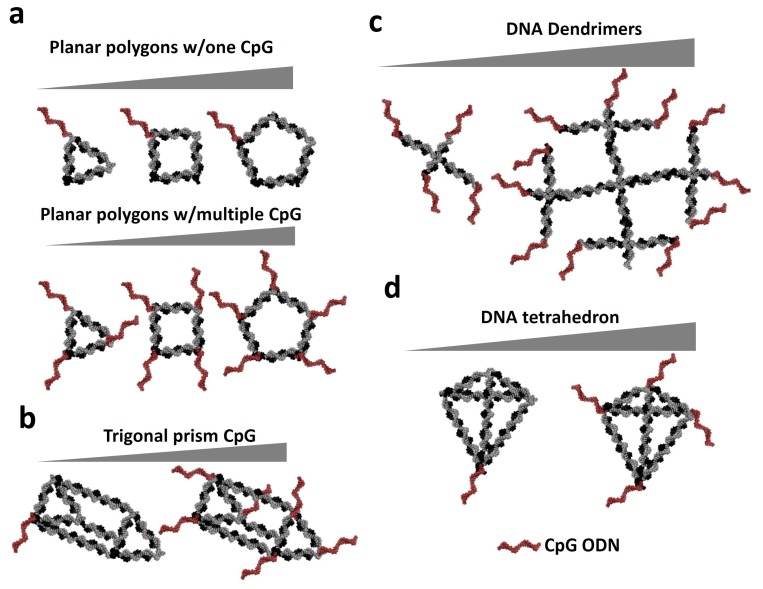
General trend of cytokine release by NANPs. The cytokine induction increase was observed by different cytosine-phosphate-guanosine (CpG) carriers including planar polygonal 2′-f modified NPs (**a**) 2′f modified RNA triangular prisms (**b**) RNA and DNA dendrimeric nanostructures (**c**) and DNA tetrahedrons (**d**).

Various studies have attempted to use CpG conjugated into NANPs to evaluate immunostimulatory efficacy as a function of nanoparticle size and shape [13,16,76,77] (Figure 4).

For example, Guo’s group utilized planar nanoparticles of triangular (~10 nm), tetragonal (~12 nm), and pentagonal (~13 nm) shapes [13]. Their study showed 2′F-modified RNA nanoparticles decorated with CpG can be readily recognized by TLR-9 receptors on the endosomal membrane of macrophages as a result of cellular uptake. The highest level of secretion of pro-inflammatory cytokines TNF-α and IL-6 was obtained with the triangle nanoparticles carrying one CpG motif when compared to the square and pentagon nanoparticles. It is believed that due to the compactness of the triangular structure, it can be more rapidly internalized into the cells as compared to the pentagon structure. However, by increasing the numbers of CpG per RNA nanoparticle, the opposite effect was found. The highest level of secretion of pro-inflammatory cytokines was attained with the pentagon nanoparticle loaded with the maximum number of CpG ODNs. In addition to planar CpG functionalized RNA nanostructures, cytokine induction can be triggered by various 3D RNA nanoarchitectures [16]. Immunostimulatory properties of triangular nanoprisms correlated with the number of CpG payload per nanocarrier. Increasing the number of CpG per nanoparticle induced a higher level of the induction of TNF-α immune response. There are several examples known that utilize DNA nanoparticles as carriers of immunostimulatory CpG. Ding’s group has developed a method to use DNA dendrimers as a delivery system for the hairpin-loop-folded CpG ODNs [76].

The study has demonstrated an enhanced and advantageous immunostimulatory effect of the hairpin CpG compared to linear forms. Assembly of multiple CpG motifs on the most branched dendrimer is highly effective in promoting the immunostimulatory activity of CpGs. Fan’s group studied application of a DNA tetrahedron as a CpG carrier to produce an immunostimulatory response. A DNA tetrahedron containing CpG can noninvasively and efficiently enter macrophages, such as RAW 264.7 cells, without the aid of polyatomic transfection agents. The combined studies—utilizing either 2′f-modified RNA or DNA nanoparticles—illustrate the importance of the size and shape of NANPs for the improvement of activity of CpG-based immune responses by innate immune systems. However, the NANP-based delivery of the CpG system required further optimization for controllable immunostimulatory effects.

### 3.2. NANPs as Stimulators of Innate Immunity.

Recent studies employed by Afonin’s group have shown that NANPs alone (without carrying imunostimulatory agents like CpG) can be used to induce an innate immune response [9,10]. Cells can recognize a specific composition of NANPs similar to how they can recognize foreign nucleic acids to produce a type I interferon (IFN) response. In their most recent study [9], a systematic approach was undertaken to identify links between the physicochemical properties of NANPs and immunological responses. In addition, their goal was to identify the cellular and molecular mechanisms that modulate immunological recognition. As probes, 25 structurally different NANPs assembled from DNA, RNA and their mixtures of various shapes, (planar, fibrous, and globular), sizes, molecular mass, and sequences were chosen. In prior cellular studies, all assembled NANPs were extensively characterized using biophysical tools, such as an electrophoretic mobility shift assay, dynamic light scattering, and UV-melting. Furthermore, visualization of the shapes and sizes was performed by atomic force microscopy. The NANPs were tested using human peripheral blood mononuclear cells (PBMCs) from healthy donors. It was found that all NANPs can stimulate IFN secretion to some extent but only when encapsulated into a delivery carrier such as Lipoofectamine (L2K). RNA cubes showed the ability to induce type I and III IFNs at levels higher than that of the positive control, CpG. RNA particles generated greater immune-responses than their DNA analogs, while globular-shaped RNAs induced the greatest immune-stimulatory properties. These results clearly indicate NANP composition and 3D shape are key factors when studying immuno-recognition.

The success of internalization of NANPs in immune cells and subsequent magnitude of IFN response correlates with physicochemical properties of NANPs. To study internalization, RNA cubes were labeled with a fluorophore dye and exposed to PBMCs overnight. The NANP-associated fluorescence was localized to the interior of the cell, suggesting NANPs are associated with phagocytic monocytes. In correlation with earlier observations, RNA cubes were also detected on the surface of smaller lymphocytes. Lyso-ID Red was the stain source of acidic vesicles involved in the endolysosomal pathway. This confirmed that NANPs are taken up via the endolysosomal pathway associated with scavenger receptors. The initiation of the IFN response occurs by endosomal TLRs. This finding also suggests that plasmacytoid dendritic cells are the main source of IFNs.

**Figure 5:**
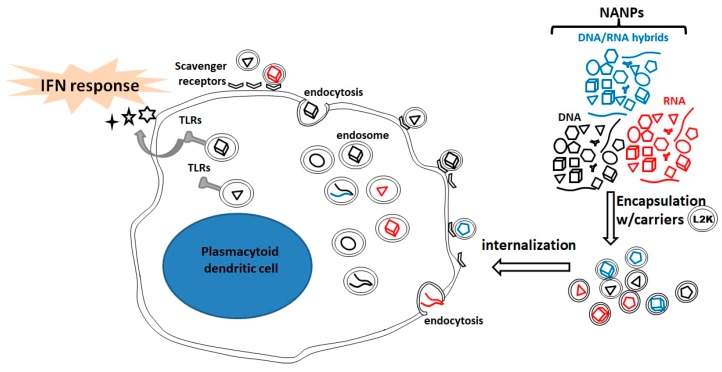
Immune response induction activated by NANPs [1]. NANPs complexed with L2K carrier first bind to cell surface scavenger receptors. The binding event is independent of NANP shape, connectivity, and sequence and does not bring about cell activation but rather endocytosis, in which endosomal toll-like receptors (TLRs) initiate the interferon (IFN) response. Upon endosomal maturation, NANPs become shape/size and sequence specifically engaged by TLRs to initiate the IFN response.

Collectively, it has been demonstrated that physiochemical characteristics including, size, 3D structure, composition and connectivity of the NANPs significantly affect immunological recognition. By using globular RNA structures, the desired induction of IFNs can be enhanced, while DNA-based NANPs, planar, and fibrous RNA structures can reduce unwanted immune responses. NANPs require delivery carriers to exhibit immunological activity irrespective of size, shape, composition, and connectivity. Through endocytosis, the NANPs enter the cells and endosomal TLRs initiate the release of IFNs primarily sourced from plasmacytoid dendritic cells (Figure 5). These findings open new doors to utilize NANPs as communication tools with the body’s immune system.

To address questions regarding specificity of TLRs to different NANPs structures, a mechanistic study involving a human peripheral blood mononuclear cell (primary immune cells) was recently explored by Hong et al. [10]. It was found that endosomal TLR7 is involved in the initiation of the interferon response to RNA cubes and rings, but not to RNA fibers nor DNA cubes. This study demonstrates shape and 3D structure of NANPs are critical features for the TLP-mediated response. However, the electroporation method to deliver NANPs needs to be avoided when performing mechanistic studies. This method affects endosomal TLR, thus impacting the IFN initiation process. This is the first study broadening our understanding of pathways of recognition of artificial NANPs by immune cells.

## 4. Conclusions

At this point, it is becoming certain that immune response can be controlled to some extent depending on the type of NANPs, and there is clearly a large library of RNA structures which can be utilized. Further understanding of mechanisms of the immune-response triggered by NANPs is critical to achieve immune induction in a controllable fashion. Although programmable NANPs offer a novel platform for controlled immune therapy, more experimental studies are necessary for progressing toward further applications in a clinical setting. Nonetheless, the first steps toward understanding recognition mechanisms of artificial NANPs by immune cells have already been taken, but there is still much to be discovered.
